# Periodic Density Functional Theory (PDFT) Predicting the Structure and Bonding Strength of Dehydrated Alkaline-Earth Metal Cation-Exchanged Chabazite Sieves (CHA-M)

**DOI:** 10.3390/molecules31132260

**Published:** 2026-06-26

**Authors:** Xiaofang Chen

**Affiliations:** Institute of Molecular Sciences and Engineering, Institute of Frontier Chemistry, School of Chemistry and Chemical Engineering, Shandong University, Qingdao 266237, China; xf.chen@sdu.edu.cn or chen_smiling@163.com

**Keywords:** periodic density functional theory (PDFT), zeolite, design, crystal structure, chemical bond, alkaline-earth metal cation site, energy gap, Bader charge, electron localization function (ELF), crystal orbital Hamilton population (COHP)

## Abstract

Ion-exchanged chabazites have wide applications in industry. In this study, dehydrated Be^2+^, Mg^2+^, Ca^2+^, Sr^2+^, and Ba^2+^-exchanged chabazite sieves (CHA-M) were designed, which differ from some reported natural and hydrated chabazite mineral series. Their crystal structures and electronic properties were carefully predicted using the PDFT//PBE+TS/HI method. Each CHA-M deviates from the space group R-3M slightly. The calculated crystal volume of CHA-M was underestimated by up to −3.28% relative to the related chabazite mineral. The alkaline-earth metal cation (M^2+^) with the Bader charge of +1.67 ~ +1.72 *e* gradually moves to the center of the 8MR window of the CHA framework as the atomic number increases. The predicted HOBO-LUBO gap of CHA-M ranges from 3.74 to 5.33 eV. The alkaline-earth metal atom serves as the primary electron-accepting site in CHA-M. The M^2+^-[CHA]^2−^ bond is assumed to have an −ICOHP value ranging from 0.327 to 4.005 eV and a BDE value ranging from 4.61 to 8.75 eV. Contrary to CHA-Mg, CHA-Be is likely to exhibit the highest HOBO-LUBO gap, the strongest bonding strength between M^2+^ and [CHA]^2−^, and the biggest absolute value of Fermi energy (i.e., −2.193 eV) among the five species. This study may help researchers to design new zeolites.

## 1. Introduction

**Scheme 1:**
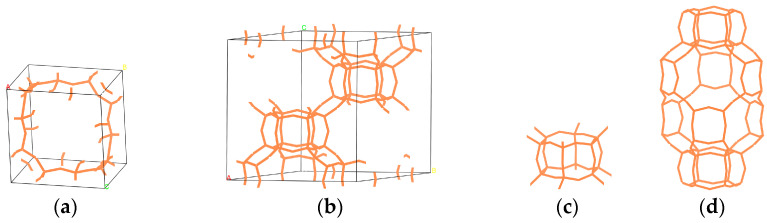
Schematic structure: (**a**) rhombohedral and (**b**) hexagonal crystal structure in the CHA framework; (**c**) a secondary building unit, the double six-membered ring (D6R) layer; and (**d**) a 8^6^6^4^4^24^ cage.

Chabazite sieves are well-known natural zeolites [1,2,3,4,5,6] with CHA topology in the space group R-3M (No. 166), exhibiting either a rhombohedral (Scheme 1a) or hexagonal representation (Scheme 1b). The CHA framework is composed of a series of secondary building units (SBUs) called the double six-membered ring (D6R) layers (Scheme 1c). Previous X-ray experiments have demonstrated that the CHA framework structure has four types of three-dimensional windows: four-membered ring (4MR), six-membered ring (6MR), eight-membered ring (8MR), and twelve-membered ring (12MR) windows (Scheme 1) [1]. Analysis of a typical 8^6^6^4^4^24^ cage (Scheme 1d) in the supercell of the CHA framework structure indicates that the peripheral 12MR window and the internal 8MR and 4MR windows are almost perpendicular to the D6R layer building units located above and below. It is commonly accepted that the 8MR window of the CHA framework is the primary region of catalysis and adsorption [7,8]. The isomorphous Si^4+^→Al^3+^ substitution and the following charge balance by a proton produces a Brönsted acid site. Generally, the acid strengths of zeolites are greatly influenced by their framework structure, Si/Al ratio, and substitution site. The unique topology and acid strength, together with their dispersion properties, enable CHA-framework zeolites (e.g., SSZ-13 and SAPO-34) to serve as catalysts [9,10,11], adsorbents [1,12,13,14,15,16], and separators [15]. For example, modified chabazites have the ability to remove heavy metal cations or pollutant anions from aqueous solutions [17].

Alkaline-earth metal elements are abundant in nature. This study aims to design the structure and predict the properties of alkaline-earth metal cation (M^2+^)-exchanged chabazite sieves ([CHA]^2−^), which are referred to as CHA-M for simplicity. Herein, the alkaline-earth metal elements considered are Be, Mg, Ca, Sr, and Ba. The van der Waals (vdW) radii are about 1.90, 2.20, 2.40, 2.55, and 2.70 Å for Be^2+^, Mg^2+^, Ca^2+^, Sr^2+^ and Ba^2+^, respectively, as reported by Batsanov [18]. These five alkaline-earth metal cations are capable of accessing the 3.8 Å × 3.8 Å 8MR window of the CHA framework. The alkaline-earth metal cation of the designed CHA-M is initially positioned in the cavity of the 8MR window of the CHA framework, without replacing any framework atoms. For more details, please refer to Section 3.

The designed CHA-M represents a new species, distinct from natural and hydrated cation-exchanged chabazite mineral series [1,2,3,4,5,6,19,20,21]. Natural chabazite mineral series typically contain a large number of water molecules coordinated to the metal cations. In natural and hydrated chabazite minerals, the metal cation tends to reside near the center of the large cage in the CHA framework. Moreover, natural chabazite mineral series typically contain a mixture of various metal cations. It is worth noting that Be has never been reported in natural crystalline chabazite mineral series in the literature, to the best of my knowledge.

Lattice parameters are fundamental physical constants of materials. The crystalline chabazite sieve has the space group R-3M (No. 166) with a lattice length (a) of 9.28 Å and a lattice angle (α) of 94.27°. Alkaline-earth metal cations would have effects on the CHA framework structure. At a minimum, they tend to reduce the window size of the initial CHA framework structure due to their occupancy. The second issue of interest is the alkaline-earth metal cation location within the designed CHA-M. The alkaline-earth metal cations in natural chabazite mineral series are surrounded by several water molecules, and both the cations and water molecules are observed to vary significantly across different forms [2,3,4,5,6,19,20,21,22]. However, the designed CHA-M does not contain any water molecules. The alkaline-earth metal cation of the designed CHA-M interacts directly with the CHA framework structure. The third issue of interest is how the alkaline-earth metal cations interact with CHA framework structure in the designed CHA-M. The alkaline-earth metals are located in Group IIA of the elemental table. Each alkaline-earth metal atom has two electrons in the outermost *s* shell, which can be shared with CHA framework oxygen atoms. It is necessary to examine the charge distribution in CHA-M. The interaction between M^2+^ and [CHA]^2−^ can likely be decomposed into the sum of interactions between alkaline-earth metals and several CHA framework oxygen atoms. The chemical bonding strength between M^2+^ and [CHA]^2−^ arises from interatomic orbital contributions, according to the theory that atomic orbitals combine linearly into crystal orbitals. The alkaline-earth metal cations are suggested to play a role in the stabilization of natural zeolites with large cage structures [23]. What is the bonding strength between M^2+^ and [CHA]^2−^ in the designed CHA-M? The last issue of interest is the property change trend of the designed CHA-M with different alkaline-earth metal cations, as the five alkaline-earth metal atoms have different tendencies to lose electrons.

This study addresses these issues using the periodic density functional theory (PDFT). The long-range dispersion interactions are corrected via the Tkatchenko–Scheffler scheme combined with iterative Hirshfeld partitioning (TS/HI) [24,25,26]. The PDFT//PBE+TS/HI method is highly accurate for the study of crystalline systems [14,27]. The energy minimization method with a three-step procedure is used to carefully simulate the crystal structure of CHA-M, followed by phonon analysis. The charge distribution of CHA-M is analyzed using the Bader charge. The highest occupied band orbital (HOBO) and the lowest unoccupied band orbital (LUBO) are used to roughly estimate the excitation energy, possible excitation sites, and the conductivity of the designed CHA-M. The electron localization function (ELF) is used to determine whether two adjacent atoms are chemically bonded. The crystal orbital Hamilton population (COHP) method is used to estimate the atomic orbital contribution, the bonding mode, and the bonding strength. The calculated BDE value of the M^2+^-[CHA]^2−^ bond will be compared with the experimental BDE value of the corresponding M-O bond in crystallographic alkaline-earth metal oxides. This study may help researchers to design structures and predict the properties of new zeolites.

## 2. Results and Discussion

Dehydrated CHA-M rather than the hydrated species was selected as the object of study, as hydration was found to lead to cation site distribution across the space, changing the CHA framework shape into different forms [2,3,4,5,6,19,20,21,22]. The initial geometry of dehydrated CHA-M was constructed from an all-silica CHA framework through two isomorphous Si^4+^→Al^3+^ substitutions within one 8MR window of an all-silica CHA framework, followed by introducing one alkaline earth metal cation (M^2+^) at the intersection of three atoms in the -Al-O-Si-O-Al-O linkage within the resulting double Al-substituted CHA framework structure. For details, please refer to Section 3. The crystal structure and the electron properties described in the following text are the results predicted under the periodic boundary condition when the K-point meshes were set to 4 ×4 × 4.

### 2.1. Crystal Structure of CHA-M

#### 2.1.1. Lattice Parameters

Table 1 shows that three crystal lengths (i.e., a = 9.41, b = 8.68, c = 9.64 Å) of CHA-Be are unequal, as are three crystal angles (i.e., 92.97°, 94.56°, and 94.98°). Accordingly, CHA-Be deviates from the space group R-3M. Previously, Smith et al. [22] determined through neutron diffraction that dehydrated HSSZ-13 has the space group R-3M, with a = 9.28 Å and α = 94.27°. Through analysis, the crystal volume of CHA-Be was found to be 11.42 Å^3^ below the single unit-cell volume of dehydrated HSSZ-13 [22]. Regarding CHA-Mg, three lattice lengths and three lattice angles fluctuate around those of dehydrated HSSZ-13 [22]. The absolute deviations of the former and latter are −0.17 ~ 0.29 Å and −0.14 ~ 0.77°, respectively. When the hydrated chabazite-Mg mineral is selected as the reference [6], the absolute deviations are −0.26 ~ 0.20 Å and −0.48 ~ 0.43° for the lattice length and lattice angle, respectively. As a result, the unit cell volume of CHA-Mg is predicted to be about 811.51 Å^3^, which is 19.19 Å^3^ higher than that of the dehydrated HSSZ-13 reported by Smith et al. [22] and 3.43 Å^3^ lower than the single crystal and hydrated chabazite-Mg reported by Montagna et al. [6]. CHA-Ca has a unit cell volume of 815.66 Å^3^, which is 5.85 or 9.94 Å^3^ smaller than the crystal volume of hydrated chabazite-Ca mineral reported by Nakatsuka et al. [19] or Smith et al. [2]. Relative to dehydrated HSSZ-13, both CHA-Sr and CHA-Ba have larger lattice lengths, with increases of about 0.02 ~ 0.20 Å and 0.05 ~ 0.12 Å, respectively. Moreover, CHA-Ba has three larger lattice angles, while CHA-Sr has two larger lattice angles. As a result, the predicted unit cell volumes of CHA-Sr and CHA-Ba deviate from those of dehydrated HSSZ-13 [22] by 21.69 and 9.32 Å^3^, while being about 5.38 and 27.14 Å^3^ lower than those of the related chabazite mineral series in reported experiments, respectively [4,5].

When the designed and dehydrated CHA-M structures are compared with dehydrated HSSZ-13 [22] and the natural and hydrated chabazite minerals chabazite-Mg [6], chabazite-Ca [19], chabazite-Sr [5], and chabazite-Ba [4], the absolute errors in the crystal volumes range from −27.14 to −3.43 Å^3^, and the percent calculation errors are within −3.27%. Table 1 also lists the mean absolute deviation (MAD) of the lattice lengths (MAD-abc) and lattice angles (MAD-αβγ), as well as the root mean square deviation (RMSD) of the lattice lengths (RMSD-abc) and lattice angles (RMSD-αβγ). For five dehydrated CHA-M species, MAD-abc ranges from −0.01 to −0.06, and MAD-αβγ ranges from −0.10 to 2.49. RMSD-abc ranges from 0.07 to 0.41, and RMSD-αβγ ranges from 0.60 to 2.53. Therefore, the calculated lattice parameters of the designed and dehydrated CHA-M are in line with the related experimental values of the natural and hydrated mineral series [4,5,6,19,22].

#### 2.1.2. Bond Length

As predicted, the Si-O, Al-O, and M-O bond distances in each CHA-M structure fluctuate within a narrow range while remaining consistent with standard bond lengths (Table 1). In CHA-Be, the Si-O bond distance ranges from 1.59 to 1.70 Å, the Al-O distance ranges from 1.71 to 1.93 Å, and the Be-O bond ranges from 1.58 to 1.63 Å. The Si-O and Al-O bond distances within CHA-Mg vary slightly with position, with the former ranging from 1.60 to 1.68 Å and the latter ranging from 1.72 to 1.86 Å. The Mg-O distance within CHA-Mg is longer than the Be-O distance within CHA-Be, ranging from 1.96 to 2.00 Å. The Si-O and Al-O distances within CHA-Ca are close to those within CHA-Mg, ranging from 1.59 to 1.66 Å and 1.72 to 1.81 Å, respectively. The Ca-O distance within CHA-Ca is significantly larger than the Mg-O distance within CHA-Mg, ranging from 2.29 to 2.30 Å. The Si-O and Al-O distances within CHA-Sr are close to those within CHA-Ba, ranging from 1.59 to 1.67 Å and from 1.71 to 1.78 Å, respectively. The Sr-O distance within CHA-Sr is 0.24~0.29 Å shorter than the Ba-O distance within CHA-Ba. Within the five CHA-M species, the Si-O distance remains almost identical, as does the Al-O distance; however, the M-O distance increases with the increasing atomic number of the alkaline-earth metal.

#### 2.1.3. Alkaline-Earth Metal Cation Location

**Figure 1:**
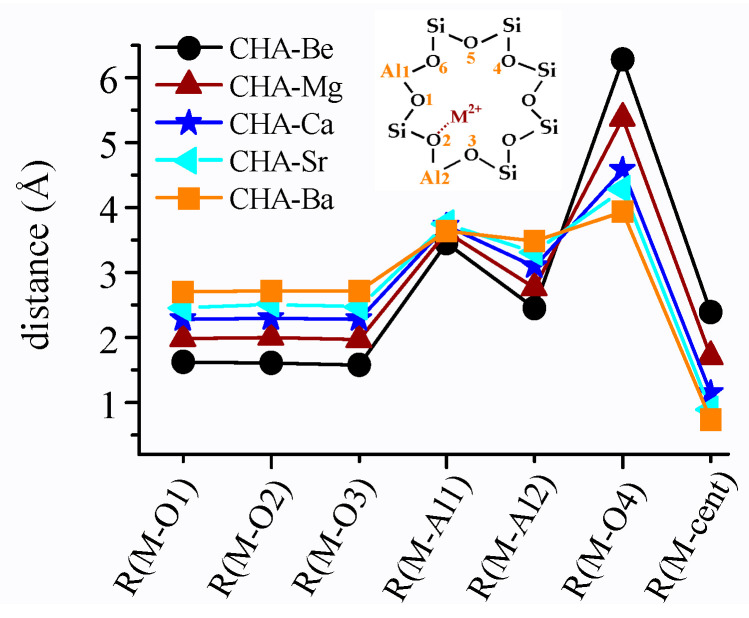
Interatomic distances (in Å) of interest in the eight-member ring (8MR) cavity of alkaline-earth metal cation-exchanged CHA structures (CHA-M). Note that the color and the atomic numbering of interest are provided in subfigure; Al atoms are in orange; Si and O atoms are in black, alkaline-earth metal cation (M^2+^) is in brown; the atomic numbering is anticlockwise.

**Figure 2:**
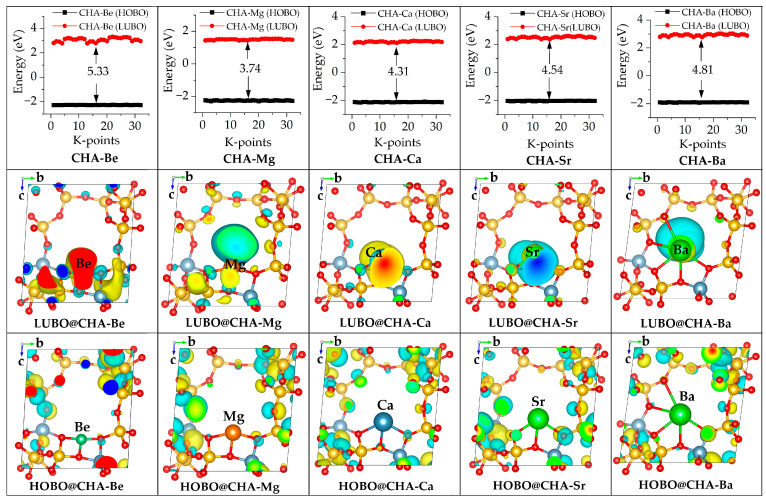
Schematic diagrams of the highest occupied band orbital (HOBO), the lowest unoccupied band orbital (LUBO), and the HOBO-LUBO gap for the designed and dehydrated alkaline-earth metal cation-exchanged chabazite sieves (CHA-M). Note that O atoms are in red; Si atoms are in yellow; Al atoms are in pastel blue; Be, Sr, and Ba atoms are in green; Mg atoms are in brown; and Ca atoms are in sky blue in the ball-and-stick model.

The metal cation location is of interest. The calculation results indicate that the alkaline-earth metal cation (M^2+^) in the designed and dehydrated CHA-M is nearly in the plane of the 8MR window of the CHA framework (Figure 1 and Figure 2). This location is different from that in the related and hydrated chabazite minerals reported in studies [4,5,6,19,22]. The main difference lies in the hydration effect on cation coordination and the CHA framework shape. The M^2+^ locations of the five CHA-M species differ slightly from one another (Figure 1 and Figure 2). Within CHA-Be, CHA-Mg, CHA-Ca, CHA-Sr, and CHA-Ba, as predicted, the M-O1 distances (i.e., R(M-O1)) are about 1.63, 1.98, 2.28, 2.45, and 2.70 Å while the M-O2 distances (i.e., R(M-O2)) are about 1.62, 2.00, 2.30, 2.53, and 2.72 Å. Both R(M-O1) and R(M-O2) increase with the increasing atomic number of the alkaline-earth metal. The distance between M^2+^ and the opposite O4 atom in the 8MR window of the CHA framework (i.e., R(M-O4)) decreases from 6.29 Å to 3.94 Å, while R(M-Al2) increases from 2.45 to 3.49 Å as M^2+^ transitions from Be^2+^ to Ba^2+^. The midpoint between O2 and O4 atoms, denoted as M(O2-O4), can be regarded as the center of the 8MR window of CHA-M. The distances between M(O2-O4) and M^2+^ are about 2.39, 1.71, 1.14, 0.88, and 0.74 Å for Be^2+^, Mg^2+^, Ca^2+^, Sr^2+^, and Ba^2+^, respectively. The declining distance between M(O2-O4) and M^2+^ implies that the metal cation gradually moves toward the center of the 8MR window of the CHA framework structure as the atomic number increases.

### 2.2. Bader Charge in CHA-M

The Bader charge of an atom is an indicator of its charge distribution. As calculated, the alkaline-earth metal cation in the designed and dehydrated CHA-M has a Bader charge ranging from +1.67 to +1.72 *e* (Table 2), which is lower than 2.00. This indicates that the two electrons on the outermost shell of the alkaline-earth metal atom do not completely transfer to the CHA framework structure. The positive charge of the alkaline-earth metal originates from electron loss, with the transferred electrons distributed to the CHA framework structure. Five CHA framework oxygen atoms adjacent to each alkaline-earth metal atom, denoted as O1, O2, O3, O5, and O6, have Bader charges ranging from −1.58 to −1.65 *e*. Within CHA-M, the alkaline-earth metal atom serving as the electron donor transfers the electron to the virtual orbital of the CHA framework structure.

### 2.3. Highest Occupied Band Orbital and Lowest Unoccupied Band Orbital

The highest occupied band orbital (HOBO) and the lowest unoccupied band orbital (LUBO) are called frontier orbitals. The electrons occupying the frontier orbitals are known as frontier electrons. The energy level of the HOBO reflects the ionization energy (i.e., the ability of a system to lose an electron), while the energy level of the LUBO reflects the electron-accepting ability (i.e., the tendency of a system to gain an electron); in particular, higher HOBO energy corresponds to a greater ability to lose electrons, while a lower LUBO level suggests a higher electron-accepting ability. A narrow HOBO-LUBO gap tends to facilitate electronic excitation and is associated with the higher activation capability.

The electron density of the HOBO in dehydrated CHA-M is delocalized over the entire CHA framework structure, while the electron density of LUBO in dehydrated CHA-M almost surrounds the alkaline-earth metal atoms (Figure 2). Therefore, the alkaline-earth metal atom of CHA-M serves as the primary site for electron acceptance. The HOBO-LUBO energy gap, ranging from 3.74 to 5.33 eV, suggests that these five designed and dehydrated CHA-M species would behave as insulators under specific conditions. Among them, CHA-Be is likely the most difficult species to excite, whereas CHA-Mg is the easiest. The declining energy gap indicates that the latter four species become more difficult to excite with the increasing atomic number.

Caution should be exercised when applying the frontier orbital theory to estimate the reactivity/activation of zeolites. This application typically requires extensive calculations to determine the precise crystal structure, the orbital location, and energy levels. The frontier orbitals of zeolites with multi-functional groups generally spread over larger portions of the framework, rather than remaining strictly localized on a single atom. In some special cases, reactivity is not determined by the frontier orbitals.

### 2.4. Chemical Bond Analysis

#### 2.4.1. Electron Localization Function

**Figure 3:**
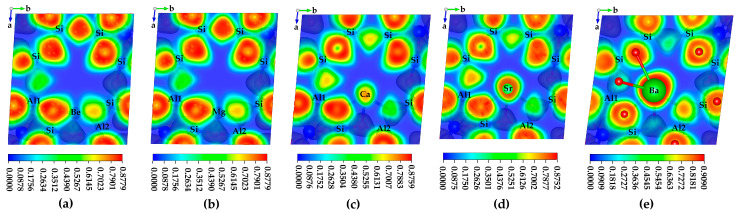
The electron localization function (ELF) of the dehydrated alkaline-earth metal cation-exchanged chabazite sieves (CHA-M) along the crystallographic [−100] plane. (**a**) CHA-Be, (**b**) CHA-Mg, (**c**) CHA-Ca, (**d**) CHA-Sr, and (**e**) CHA-Ba.

The electron localization function (ELF) can be used to estimate the classification of chemical bonds [28]. A bigger ELF value indicates a higher degree of electron localization. Figure 3 shows the electron localization function of CHA-M along the crystal plane with Miller indices of [−100]. According to the color bar below each subfigure, green corresponds to an ELF value of about 0.500. Therefore, the green zone of CHA-M suggests that a chemical bond has already formed between two adjacent atoms. As shown in the color bar, the redder zones have the higher degree of localized electrons. The yellow and red zones without an atomic label correspond to oxygen atoms. The oxygen atoms with shadows represent CHA framework O atoms located behind the [−100] crystal plane. As shown, Be, Mg, Ca, and Sr atoms chemically bind to O1, O2, and O3 atoms, while Ba atoms share electrons with five framework O atoms (i.e., O1, O2, O3, O5, and O6). For the atomic numbering, please refer to the subfigure within Figure 1. The Be and Mg atoms, shown in dark blue, have ELF values of about 0.047 and 0.019, respectively, indicating a low degree of electron localization. The Ca, Sr, and Ba atoms, shown in red, have higher electron localization, with ELF values of approximately 0.750, 0.852, and 0.894, respectively.

#### 2.4.2. Crystal Orbital Hamilton Population (COHP)

Dronskowski, R et al. [29,30,31,32,33] developed the crystal orbital Hamilton population (COHP) method. The COHP method is capable of predicting bonding mode and bonding strength and has been widely successful in the study of materials [34,35,36,37,38,39]. Bonding interactions tend to lower the energy of the system, while anti-bonding interactions increase it. As a result, the sign of the −COHP value serves as an indicator of the bonding mode. A positive −COHP value indicates a bonding interaction, whereas a negative value corresponds to an anti-bonding interaction. The −COHP value approaches zero when two atoms exhibit non-bonding interactions. The integrated crystal orbital Hamilton population (ICOHP) is an energy-resolved bonding descriptor that quantifies the bonding strength. The bonding strength of a system exhibits a positive correlation with the −ICOHP value.

**Figure 4:**
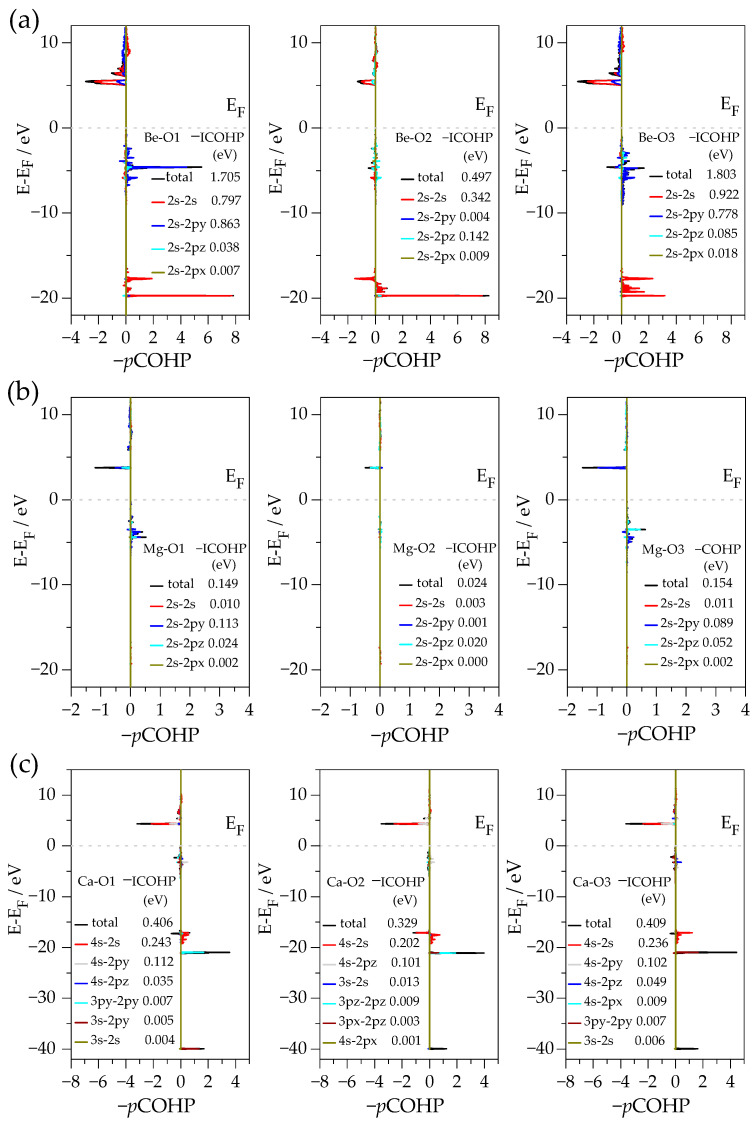

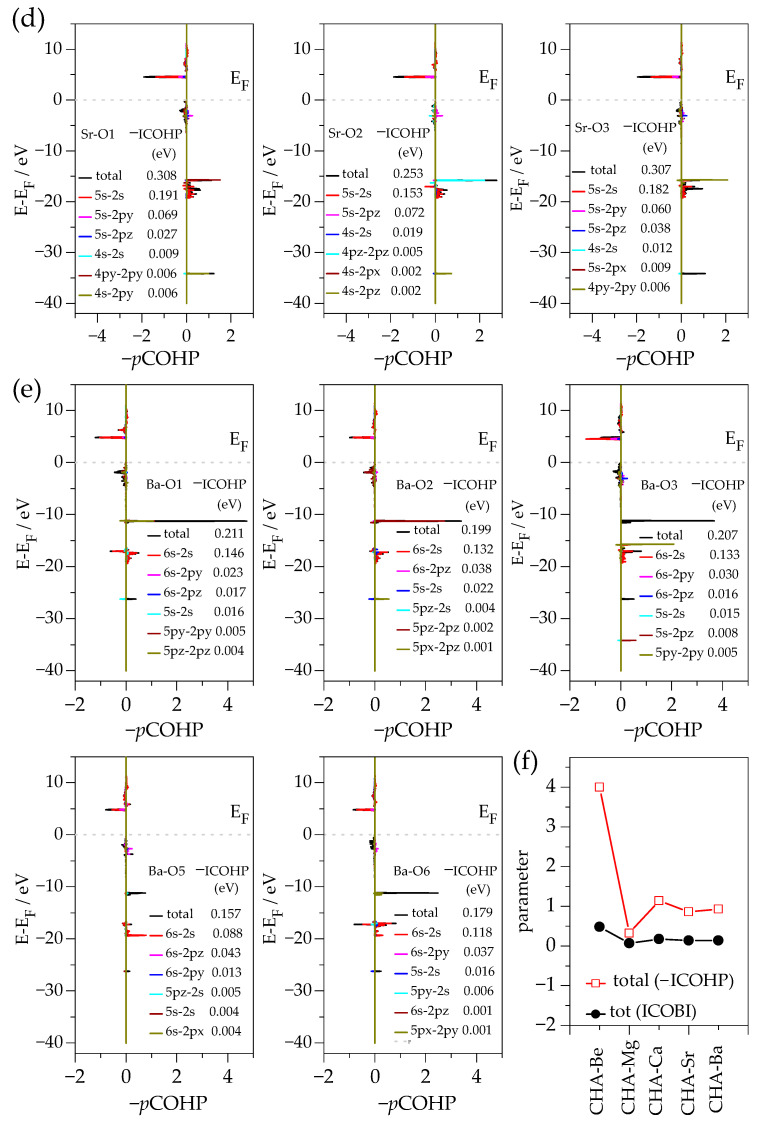
The energy (E-E_F_)-dependent crystal orbital Hamilton population (−*p*COHP) between the alkaline-earth metal atom (M) and the adjacent CHA framework oxygen atoms in the designed and dehydrated CHA-M structures: (**a**) Be-O interaction in CHA-Be; (**b**) Mg-O interaction in CHA-Mg; (**c**) Ca-O interaction in CHA-Ca; (**d**) Sr-O interaction in CHA-Sr, (**e**) Ba-O interaction in CHA-Ba. (**f**) The total integral crystal orbital Hamilton population (ICOHP) and the integral crystal bond index (ICOBI) between M and CHA framework O atoms.

The energy in Figure 4 has been adjusted to the Fermi level (E_F_) of the designed CHA-M, which decreases with increasing atomic numbering (Table 2). Figure 4a–d present three subfigures exhibiting M-Ox (M = Be, Mg, Ca, and Sr; x = 1, 2, 3) interactions while Figure 4e presents five subfigures exhibiting Ba-Ox (x = 1, 2, 3, 5 and 6) interactions. In Figure 4a–e, the bonding regime is characterized by positive −COHP values on the right side, while the anti-bonding regime is characterized by negative −COHP values on the left side. In the following text, the sharp peak refers to the bonding peak with a larger −COHP value on the right of Figure 4.

***Be-Ox interaction (x = 1, 2, and 3) in CHA-Be.*** As shown in Figure 4a, the Be-O1 interaction within CHA-Be predominantly arises from the contributions of Be[2s]-O1[2py] and Be[2s]-O1[2s] orbitals, corresponding to the blue sharp peak at around E-E_F_ = −4.603 and the red sharp peak at around E-E_F_ = −19.711 eV, respectively. The blue and red sharp peaks have −ICOHP and −COHP values of 0.863 eV and 5.497 and 0.797 eV and 7.839, respectively. The cyan and dark yellow peaks are too weak to contribute significantly to the overall Be-O1 bonding. The Be-O2 interaction in CHA-Be predominantly arises from the contribution of the Be[2s]-O2[2s] orbital, corresponding to the red sharp peaks at around E-E_F_ = −19.711 eV, with the largest −COHP or −ICOHP values of 8.252 and 0.342 eV, respectively. The secondary contribution to Be-O2 interaction arises from the Be[2s]-O2[2pz] orbital with a −ICOHP value of 0.142 eV, corresponding to a series of low cyan peaks at around E-E_F_ = −9.105 ~ 0.000 eV. The Be-O3 interaction in CHA-Be predominantly arises from the contributions of the Be[2s]-O3[2s] and Be[2s]-O3[2py] orbitals, which are associated with the red peaks at around E-E_F_ = −19.261 ~ −16.459 eV and the blue peaks at around E-E_F_ = −9.105 ~ −0.000 eV, respectively. These red and blue peaks have −ICOHP values of 0.922 eV and 0.778 eV and corresponding maximum −COHP values of 3.069 and 1.633, respectively.

The Be-O1, Be-O2, and Be-O3 interactions in CHA-Be have total −ICOHP values of 1.705, 0.497, and 1.803 eV respectively, indicating that Be^2+^ is predominantly bonded to the O3 and O1 atoms. These three Be-O bonding interactions are cumulative and collectively constitute the Be^2+^-[CHA]^2−^ bonding interaction, yielding a total −ICOHP value of 4.005 eV. This larger −ICOHP value indicates that the Be^2+^-[CHA]^2−^ bonding interaction in CHA-Be is covalent-like and strong.

***Mg-Ox interaction (x = 1, 2, and 3) in CHA-Mg.*** The −COHP peak intensity of the Mg-O interaction (Figure 4b) is substantially lower than that of the Be-O interaction (Figure 4a). The Mg-O1 bonding interaction predominantly arises from the contribution of the Mg[2s]-O1[2py] orbital, corresponding to the blue peaks at around E-E_F_ = −4.402 ~ −2.001 eV, with a maximum −ICOHP value of 0.113 eV. The lower −ICOHP values (i.e., 0.024 eV and 0.010 eV) indicate that the Mg[2s]-O1[2pz] and Mg[2s]-O1[2s] orbitals contribute less to the Mg-O1 bonding interaction. The Mg-O2 bonding interaction mainly arises from the contribution of the Mg[2s]-O2[2pz] orbital, corresponding to several low cyan peaks, yet it has a very low total −ICOHP value (i.e., 0.024 eV). The Mg-O3 bonding interaction mainly arises from the contributions of the Mg[2s]-O3[2py] and Mg[2s]-O3[2pz] orbitals. These three Mg-O bonding interactions are cumulative and collectively constitute the Mg^2+^-[CHA]^2−^ bonding interaction, yielding a total −ICOHP value of 0.327 eV. This −ICOHP value, well below 1.000 eV, suggests that the Mg^2+^-[CHA]^2−^ bonding interaction is very weak.

***Ca-Ox interaction (x = 1, 2, and 3) in CHA-Ca.*** The −COHP peak intensity of the Ca-O interaction (Figure 4c) is clearly lower than that of the Be-O interaction (Figure 4a). Over half of the Ca-O bonding interaction in the designed CHA-Ca originates from the contribution of the Ca[4s]-O[2s] orbital and corresponds to a series of red broad peaks at E-E_F_ = −16.697 ~ −18.410 eV in Figure 4c. The secondary orbital contribution to Ca-Ox bonding interaction arises from the orbital of Ca[4s]-Ox[2p] (x = 1, 2, 3), where for x = 1 and 3, 2p refers to 2py; and for x = 2, 2p refers to 2pz. However, the atomic orbital associated with the sharpest bonding peak in each subfigure in Figure 4c plays a negligible role in the Ca-O bonding interaction due to the extremely low −ICOHP value.

The Ca-O3, Ca-O1, and Ca-O2 interactions have decreasing−ICOHP values of about 0.409, 0.406, and 0.329 eV, respectively, although their differences are small. These three Ca-O bonding interactions are cumulative and collectively constitute the Ca^2+^-[CHA]^2−^ bonding interaction, yielding a −ICOHP value of 1.144 eV. This −ICOHP value, higher than 1.000 eV, suggests that the Ca^2+^-[CHA]^2−^ bonding interaction has medium strength.

***Sr-Ox interaction (x = 1, 2, and 3) in CHA-Sr.*** Analogous to the Ca-O bonding interaction, over half of the Sr-O bonding interaction in the designed CHA-Sr also originates from the contribution of the Sr[5s]-O[2s] orbital, corresponding to a series of red broad peaks at E-E_F_ = −19.261 ~ −15.809 eV in Figure 4d. The secondary orbital contribution to the Sr-Ox (x = 1, 2, 3) bonding interaction arises from the Sr[5s]-Ox[2p] (x = 1, 2, 3) orbital in magenta, where for x = 1 and 3, 2p refers to 2py; and for x = 2, 2p refers to 2pz. However, the atomic orbital associated with the sharpest bonding peak in each subfigure in Figure 4d plays a negligible role in the Sr-O bonding interaction due to the extremely low −ICOHP value.

The Sr-O1 and Sr-O3 bonds have nearly identical bonding strength based on theirsimilar −ICOHP values, both of which are slightly stronger than that of the Sr-O2 bond. These three Sr-O bonding interactions are cumulative and collectively constitute the Sr^2+^-[CHA]^2−^ bonding interaction, yielding a total −ICOHP value of 0.868 eV. This −ICOHP value, slightly lower than 1.000 eV, suggests that the Sr^2+^-[CHA]^2−^ bonding interaction has medium strength.

***Ba-Ox interaction (x = 1, 2, 3, 5, and 6) in CHA-Ba.*** Over half of the Ba-O bonding interaction of the designed CHA-Ba arises from the contribution of the Ba[6s]-O[2s] orbital, corresponding to the red broad peaks at around E-E_F_ = −19.361 ~ −16.459 eV in Figure 4e. The secondary orbital contribution to the Ba-Ox bonding interaction arises from the orbital of Ba[6s]-Ox[2p] in magenta (x = 1, 2, 3, 5, and 6), where for x = 1, 3, and 5, 2p refers to 2py; and for x = 2 and 4, 2p refers to 2pz. The atomic orbital associated with the sharpest bonding peak in the former three subfigures in Figure 4e plays a negligible role in the Ba-O bonding interaction due to the extremely low −ICOHP value.

The bonding strength decreases in the order of Ba-O1, Ba-O3, Ba-O2, Ba-O6, and Ba-O5, based on the −ICOHP values. These five Sr-O bonding interactions are cumulative and collectively constitute the Ba^2+^-[CHA]^2−^ bonding interaction, yielding a total −ICOHP value of 0.955 eV. This −ICOHP value, slightly lower than 1.000 eV, suggests that the Ba^2+^-[CHA]^2−^ bonding interaction has medium strength.

***Summary***. The −ICOHP value between the alkaline-earth metal cation (M^2+^) and the CHA framework structure anion ([CHA]^2−^) is obtained as a double summation, first over all atomic shell orbital interactions within each M-Ox interaction, and second over all M-Ox interactions. The atomic orbitals that contribute most significantly to the M-Ox bonding interaction are the outermost *s* and *p* orbitals of the M and O atoms. The M^2+^-[CHA]^2−^ bonding interactions have −ICOHP values ranging from 0.327 to 4.050 eV (Figure 4f). Among these, the Be^2+^-[CHA]^2−^ bond is the strongest and is similar to a covalent bond. The Mg^2+^-[CHA]^2−^ bond is the weakest. The Ca^2+^-[CHA]^2−^, Sr^2+^-[CHA]^2−^, and Ba^2+^-[CHA]^2−^ bonding strengths are similar to one another. The ICOBI values decrease in the order of CHA-Be, CHA-Ca, CHA-Ba, CHA-Sr, and CHA-Mg.

#### 2.4.3. Bond Dissociation Energy (BDE)

The concept of bond dissociation energy (BDE) plays a crucial role in understanding bond energy and formation energy, being helpful in quantifying the strength of chemical bonds and predicting the stability of systems. The bond dissociation energy is the minimum energy required to break a specific bond in one system.

**Figure 5:**
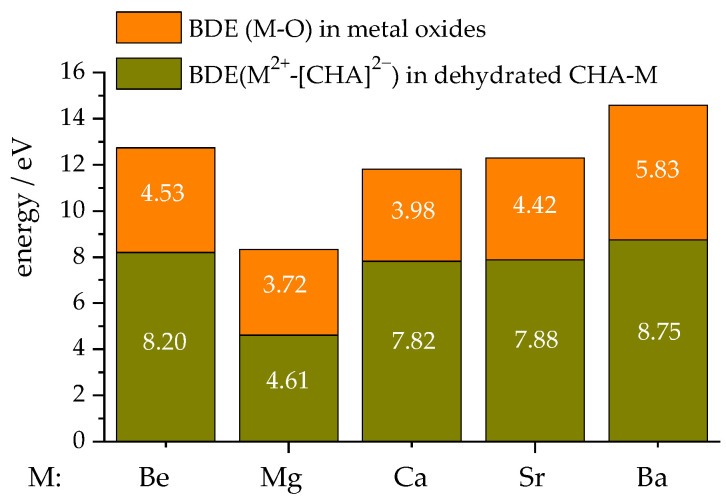
Calculated bond dissociation energy (BDE) between two fragments of alkaline-earth metal cation (M^2+^) and CHA framework structure ([CHA]^2−^) in the designed and dehydrated CHA-M. Note that BDE(M-O) refers to the BDE value between the alkaline-earth metal (M) and O atoms in the crystallographic alkaline-earth metal oxides reported in the CRC Handbook of Chemistry and Physics (95^th^ Edition (2014–2015)) [40].

In this study, the specific bond of interest in the designed CHA-M only refers to the chemical bond between the alkaline-earth metal cation (M^2+^) and the CHA framework ([CHA]^2−^), denoted as the M^2+^-[CHA]^2−^ bond. The corresponding BDE value is denoted as BDE(M^2+^-[CHA]^2−^). The BDE(M^2+^-[CHA]^2−^) value was calculated under the periodic boundary condition (PBC), on the basis of the validated crystal structure of dehydrated CHA-M. Among five BDE(M^2+^-[CHA]^2−^) values, BDE(Mg^2+^-[CHA]^2−^) is the lowest (i.e., 4.61 eV), while BDE(Ba^2+^-[CHA]^2−^) is the highest (i.e., 8.75 eV), followed by BDE(Be^2+^-[CHA]^2−^) (i.e., 8.20 eV). BDE(Ca^2+^-[CHA]^2−^) and BDE(Sr^2+^-[CHA]^2−^) are almost identical (Figure 5).

As the M-O interaction plays the crucial role in the M^2+^-[CHA]^2−^ bond of the designed CHA-M, crystallographic alkaline-earth metal oxides, which contain abundant M-O interactions, were selected as the reference for comparison with the designed CHA-M in this study. The specific bond of interest in oxides refers to the chemical bond between the alkaline-earth metal (M) and oxygen (O) atoms, whose BDE value is denoted as BDE(M-O). The BDE(M-O) value in Figure 5 was obtained from the CRC Handbook of Chemistry and Physics (95^th^ Edition (2014–2015)) [40]. With the increasing atomic number of the alkaline-earth metals, the BDE(M^2+^-[CHA]^2−^) values of the designed and dehydrated CHA-M vary in a similar manner to the BDE(M-O) values of alkaline-earth metal oxides. However, the former is about 0.89 ~ 3.84 eV higher than the corresponding value of the latter. This indicates that the M^2+^-[CHA]^2−^ bond of dehydrated CHA-M is stronger than the M-O bond in metal oxides. These conclusions should be interpreted with caution, as bonding strengths are greatly influenced by different coordination environments and bonding situations.

## 3. Materials and Methods

**Scheme 2:**
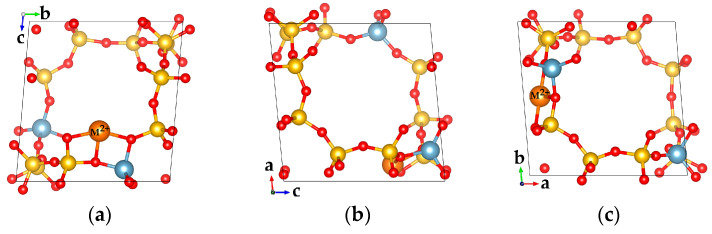
The computational structural mode of CHA-M along three crystallographic planes: (**a**) [100], (**b**) [010], and (**c**) [001]. Note that O atoms are in red, Si atoms are in yellow, Al atoms are in pastel blue, and alkaline-earth metal atoms are in brown.

The all-silica CHA framework was obtained from the Database of Zeolite Structures in Internal Zeolite Association (IZA) [41], the rhombohedral representation of which was used as the starting building structure to construct the initial geometry of dehydrated CHA-M. First, isomorphous substitution of Si^4+^ by Al^3+^ was performed twice within one 8MR window of the CHA framework to produce a double Al-substituted CHA framework structure with an -Al-O-Si-O-Al-O linkage. Then, one alkaline-earth metal cation (M^2+^) was introduced to the intersection of three O atoms in the -Al-O-Si-O-Al-O linkage within the double Al-substituted CHA framework structure to balance the charge (Scheme 2a). The initial geometry of dehydrated CHA-M was finally produced from the all-silica CHA framework structure in its rhombohedral representation. Scheme 2 illustrates the computational structural model of dehydrated CHA-M along three crystallographic planes [100], [010] and [001], with a chemical formula of Si_10_Al_2_O_24_M_1_ per unit cell.

Previous experiments have shown that, in natural and hydrated alkaline-earth metal cation-exchanged chabazite mineral series, hydration causes significant variation in both cation and water molecule sites across different forms, and the alkaline-earth metal cation tends to occupy the location near the center of the large cage [4,5,6,19,22]. I previously attempted to construct an initial guess geometry of CHA-M, in which the M^2+^ cation is near the center of the 12MR window of the CHA framework structure. Phonon analysis of the converged fully optimized geometry of dehydrated CHA-M indicated the presence of three large imaginary frequencies with absolute values exceeding 60 cm^−1^, despite many efforts to eliminate them. After failing several times, I finally selected the CHA framework containing the alkaline-earth metal cation at the intersection of three O atoms in the -Al-O-Si-O-Al-O linkage within the 8MR window as the initial guess geometry of dehydrated CHA-M (Scheme 2).

All calculations were carried out using the Vienna Ab initio Simulation Packages (VASP) [42,43,44]. The projector-augmented-wave (PAW) Perdew–Burke–Ernzerhof (PBE) pseudopotentials [45] (version 54) were employed during the plane-wave periodic gradient corrected density functional theory (DFT) calculation. To improve precision, the Tkatchenko–Scheffler scheme, in conjunction with iterative Hirshfeld partitioning (TS/HI) [24,25,26], was used to simulate long-range dispersion interactions. Many studies have demonstrated that the PDFT//PBE+TS/HI method is successful in studying crystalline systems [27], as well as many zeolitic systems [14]. The computation nodes used in this study were the same as those used in our previous study [46].

The lattice parameters of CHA-M were predicted through a three-step procedure consisting of an E-V curve scan, E-V curve fit, and subsequent full geometry optimization. The E-V curve scan was obtained through single-point energy calculations (E(V)) at specific cell volumes (V), where the volume ranged from 750 Å^3^ to 900 Å^3^ at intervals of 10 Å^3^ (Figure S1). The resulting E-V curve was then fitted using the Birch–Murnaghan equation, providing a rough estimate of the crystal structure of CHA-M. The resulting estimated crystal structure was used as the starting geometry for the following full geometry optimization. Except for CHA-Be, the energy cutoff was set to 570 eV, with the convergence thresholds of 1 × 10^−2^ eV/Å for atomic forces and 1 × 10^−7^ eV for the energy. It is difficult for CHA-Be to satisfy the convergence threshold of 1 × 10^−2^ eV/Å for atomic force. Regarding CHA-Be, the energy cutoff was set to 570 eV, with convergence thresholds of 3 × 10^−2^ eV/Å for atomic forces and 1 × 10^−7^ eV for the energy. The selected K-point meshes were gamma for the E-V scan and 4 × 4 × 4 for the full geometry optimization and property prediction to sample the Brillouin zone of CHA-M. Phonon analysis of the optimized geometry of CHA-M indicated that each CHA-M had 108 positive frequencies and 3 imaginary frequencies. Fortunately, the absolute values of the three imaginary frequencies were very low, all below 7.725 cm^−1^. These results indicate that the simulated CHA-M corresponds to an energy minimum point with the low calculation error. The charge distribution was estimated using the Bader charge, and the electron localization function was further calculated and then analyzed along the [−100] crystallographic plane of CHA-M.

The chemical bonding between the alkaline-earth metal atom (M) and the adjacent CHA framework oxygen atom (O) was analyzed using the crystal orbital Hamilton population (COHP) method in this study. The COHP method based on density functional theory is a useful tool to predict bonding modes and bonding strengths in periodic systems [29,30,31,32,33]. In COHP theory, the band energy (*E^bond^*) is expressed in terms of the crystal orbital Hamilton population ($H_{R{LR}^{\prime }L^{\prime }}$) and the density matrix ($n_{R{LR}^{\prime }L^{\prime }}$) (Equation (1)), while the energy (ϵ) integration of the density of state (*DOS*) matrix ($N_{R{LR}^{\prime }L^{\prime }}(\epsilon )$) yields the density matrix (Equation (2)).

(1) $$E^{bond}=\sum_{R}\sum_{L}\sum_{R^{\prime }}\sum_{L^{\prime }}H_{R{LR}^{\prime }L^{\prime }}\cdot n_{R{LR}^{\prime }L^{\prime }}$$

(2) $$n_{R{LR}^{\prime }L^{\prime }}=\int^{E_{F}}d\epsilon \cdot N_{R{LR}^{\prime }L^{\prime }}\left(\epsilon \right)$$

Herein, *R* and *R*′ refer to the first atom site and the second atom site, respectively, *L* and *L*′ refer to the first angular momentum quantum numbers *L* = *lm* and the second angular momentum quantum numbers *L*′ = *l*′*m*′, respectively. *E_F_* is the Fermi energy level. The positive or negative sign of the off-diagonal COHP indicates whether two atoms are bonded: (1) negative COHP values correspond to bonding interactions that lower the system’s energy, and (2) positive COHP values correspond to anti-bonding interactions, which increase the system’s energy. The magnitude of the integrated COHP value (ICOHP) indicates the bonding strength.

The bond dissociation energy between M^2+^ and [CHA]^2−^ was calculated as the energy difference between M^2+^ plus [CHA]^2−^ and CHA-M. The geometries of both M^2+^ and [CHA]^2−^ were extracted directly from the converged optimization geometry of CHA-M with K-points of 4 × 4 × 4. The charge of M^2+^ was set to +2 and the charge of [CHA]^2−^ was set to −2 during the BDE calculation.

## 4. Conclusions

In this study, five new species of CHA-M were designed by introducing Be^2+^, Mg^2+^, Ca^2+^, Sr^2+^, and Ba^2+^ cations to the cavity of an 8MR window of the CHA framework structure in the rhombohedral representation. To the best of my knowledge, CHA-Be is reported here for the first time. The remaining four are also new species and are clearly distinct from the previously reported chabazite-Mg, chabazite-Ca, chabazite-Sr, and chabazite-Ba minerals, respectively [1,2,3,4,5,6,19,20,21]. The designed CHA-M belongs to dehydrated species, in which Be^2+^, Mg^2+^, Ca^2+^, Sr^2+^, or Ba^2+^ is chemically bonded to CHA framework oxygen atoms. However, the chabazite mineral series belong to hydrated species, in which the alkaline-earth metal cation is coordinated to multiple water molecules, and hydration leads to spatially distributed metal cations and water molecules. The first novelty of this study is the structural design of new zeolites. The second novelty is the determination of crystal structures and subsequent prediction of electronic properties of previously unknown zeolites, carried out using the highly accurate PDFT//PBE+TS/HI method. The third novelty of this study is the investigation into the change trends in crystal structure, metal cation site location, and electronic properties of the five designed CHA-M systems with increasing atomic number of the alkaline-earth metals.

The crystal structures of the designed CHA-M available in this study are reliable and exhibit low calculation error. Each CHA-M deviates slightly from the space group R-3M, and their predicted crystal volumes are underestimated by up to −3.27% relative to the related natural chabazite minerals. The alkaline-earth metal cation in CHA-M, with a Bader charge of +1.67 ~ +1.72 *e*, gradually moves toward the center of the 8MR window of the CHA framework structure as the atomic number increases. The five dehydrated CHA-M species likely behave as insulators under specific conditions; among them, CHA-Be is likely the most difficult to excite, whereas CHA-Mg is the easiest.

ELF analysis indicated that Be^2+^, Mg^2+^, Ca^2+^, and Sr^2+^ are all chemically bonded to three CHA framework O atoms (i.e., Ox (x = 1, 2, and 3)), while Ba^2+^ is bonded to five CHA framework O atoms (i.e., Ox (x = 1, 2, 3, 5, and 6)). The crystal orbital Hamilton population analysis of CHA-M allows the following conclusions to be drawn:(1) The atomic orbitals contributing most significantly to the M-Ox bonding interaction are the outermost *s* and *p* orbitals of the alkaline-earth metal and oxygen atoms. (2) The bonding interaction between M^2+^ and [CHA]^2−^ can be regarded as the sum of the chemical bonding interactions between M^2+^ and all bonded CHA framework Ox atoms. (3) Among the five M^2+^-[CHA]^2−^ bonding interactions, the Be^2+^-[CHA]^2−^ interaction is the strongest; the Mg^2+^-[CHA]^2−^ interaction is the weakest. The Ca^2+^-[CHA]^2−^ interaction is the second strongest, followed by the Sr^2+^-[CHA]^2−^ and Ba^2+^-[CHA]^2−^ interactions. The BDE(M^2+^-CHA]^2−^) values of the designed CHA-M are about 0.89 ~ 3.84 eV higher than the corresponding BDE(M-O) values of the crystallographic alkaline-earth metal oxides reported in a previous study [40]. This indicates that M^2+^ bonded to oxygen atoms in the CHA framework are stronger than that bonded to O atoms in crystallographic alkaline-earth metal oxides. The higher values of both −ICOHP and BDE between M^2+^ and [CHA]^2−^ are important indicators of the structural stability of CHA-M [23]. The conclusions regarding relative bonding strengths should be interpreted with caution, as bonding strengths are greatly influenced by different coordination environments and bonding situations.

This study helps to explain the nature of alkaline-earth metal cation-exchanged zeolites, and may even assist in designing structures and predicting the properties of new zeolites.

## Figures and Tables

**Table 1:** Lattice parameters calculated using the PDFT//PBE+TS/HI method when the K-point meshes of CHA-M are set to 4 × 4 × 4.

Parameters	CHA-Be	CHA-Mg	CHA-Ca	CHA-Sr	CHA-Ba
a (Å)	9.41	9.40	9.33	9.31	9.35
b (Å)	8.68	9.11	9.29	9.30	9.33
c (Å)	9.64	9.57	9.51	9.48	9.40
α (°)	92.97	94.35	94.54	94.47	96.64
β (°)	94.56	95.04	94.79	94.79	97.30
γ (°)	94.98	94.13	93.96	93.40	96.15
V (Å^3^)	780.90	811.51	815.66	814.01	801.64
ΔV1 (Å^3^)	−11.42	19.19	23.34	21.69	9.32
ΔV2 (Å^3^)	−11.42	−3.43	−5.85	−5.38	−27.14
−%(ΔV)	1.44%	0.42%	0.71%	0.66%	3.27%
MAD-abc (Å)	−0.04	−0.01	−0.01	−0.02	−0.06
MAD-αβγ (°)	−0.10	−0.10	0.05	0.28	2.49
RMSD-abc	0.41	0.19	0.10	0.08	0.07
RMSD-αβγ	0.60	0.40	0.35	0.66	2.53
Si-O distance (Å)	1.59–1.70	1.60–1.68	1.59–1.66	1.59–1.67	1.59–1.66
Al-O distance (Å)	1.71–1.93	1.72–1.86	1.72–1.81	1.71–1.78	1.71–1.78
M-O distance (Å)	1.58–1.63	1.96–2.00	2.29–2.30	2.46–2.53	2.70–2.82

Notes: (1) Dehydrated HSSZ-13 [22], and the natural and hydrated chabazite minerals chabazite-Mg [6], chabazite-Ca [19], chabazite-Sr [5], and chabazite-Ba [4] were selected as the reference species for the designed and dehydrated CHA-Be, CHA-Mg, CHA-Ca, CHA-Sr, and CHA-Ba, respectively. (2) MAD-abc and MAD-αβγ represent the mean absolute deviation (MAD) of the predicted lattice lengths and lattice angles of the designed dehydrated CHA-M in this work relative to the reference species [4,5,6,19,22]. (3) RMSD-abc and RMSD-αβγ represent the root mean square deviation (RMSD) of the predicted lattice lengths and lattice angles of the designed and dehydrated CHA-M in this work relative to the reference species [4,5,6,19,22]. (4) ΔV1 (or ΔV2) refers to the crystal volume difference between the predicted value in this work and the experimental value of the dehydrated HSSZ-13 species [22] (or the reference species [4,5,6,19,22]). (5) %(ΔV) is equal to ΔV2 divided by the crystal volume of the reference species [4,5,6,19,22].

**Table 2:** The Bader charge (a.u.) of alkaline-earth metal atom (M) and the adjacent CHA framework oxygen atoms, as well as the Fermi energy (E_F_) of the designed and dehydrated CHA-M, calculated by the PDFT//PBE+TS/HI method when the K-point samples are set to 4 × 4 × 4.

Item		CHA-Be	CHA-Mg	CHA-Ca	CHA-Sr	CHA-Ba
Bader charge (e) in CHA-M	M	+1.72	+1.71	+1.67	+1.72	+1.72
	O1	−1.65	−1.62	−1.60	−1.60	−1.61
	O2	−1.63	−1.61	−1.58	−1.59	−1.59
	O3	−1.65	−1.61	−1.59	−1.60	−1.60
	O5	−1.59	−1.60	−1.61	−1.61	−1.61
	O6	−1.59	−1.60	−1.60	−1.61	−1.60
E_F_ (eV)		−2.19	−2.09	−2.01	−1.87	−1.83

## Data Availability

The original contributions presented in this study are included in the article/Supplementary Material. Further inquiries can be directed to the corresponding author.

## Supplementary Materials

The following supporting information can be downloaded at https://www.mdpi.com/article/10.3390/molecules31132260/s1: Figure S1: The total energy dependent on the lattice volume of the designed and dehydrated alkaline-earth metal cation-exchanged chabazite sieves, Table S1: The fractional coordinates of CHA-Be, CHA-Mg, CHA-Ca, CHA-Sr, and CHA-Ba.

## Abbreviations

The following abbreviations are used in this manuscript:

BDE	bond dissociation energy
CHA	chabazite
CHA-M	dehydrated alkaline-earth metal cation-exchanged chabazite sieves
COHP	crystal orbital Hamilton population
DMTO	dimethyl ether/methanol into light olefins
DOS	density of state
D6R	double six-membered rings
ELF	electron localization function
HOBO	highest occupied band orbital
ICOHP	integrated crystal orbital Hamilton population
ICOBI	integral crystal bond index
IZA	Internal Zeolite Association
LUBO	lowest unoccupied band orbital
MAD-abc	mean absolute deviation of lattice length
MAD-αβγ	mean absolute deviation of lattice angle
M	alkaline-earth metal
PAW	projector-augmented-wave
PBE	Perdew–Burke–Ernzerhof
−pCOHP	opposite values of the crystal orbital Hamilton population
PDFT	periodic density functional theory
RMSD-abc	root mean square deviation of lattice length
RMSD-αβγ	root mean square deviation of lattice angle
SBU	secondary building unit
TS/HI	Tkatchenko–Scheffler scheme combined with iterative Hirshfeld partitioning
vdW	van der Waals
4MR	four-membered ring
6MR	six-membered ring
8MR	eight-membered ring
12MR	twelve-membered ring
VASP	Vienna Ab initio Simulation Packages
