# Supplementary material for: Periodic Density Functional Theory (PDFT) Predicting the Structure and Bonding Strength of Dehydrated Alkaline-Earth Metal Cation-Exchanged Chabazite Sieves (CHA-M)
Source: Molecules. 2026 Jun 26;31(13):2260. doi: 10.3390/molecules31132260 (PMC13363160; doi:10.3390/molecules31132260)
Supplement: Supplementary file 1 [file molecules-31-02260-s001.zip › molecules-3997625-supplementary.pdf]

Supplementary Materials for Article

Periodic Density Functional Theory (PDFT) Predicting the Structure and Bonding Strength of Dehydrated Alkaline-Earth Metal Cation-Exchanged Chabazite Sieves (CHA-M)

Xiaofang Chen

Institute of Molecular Sciences and Engineering, Institute of Frontier Chemistry, School of Chemistry and Chemical Engineering, Shandong University, Qingdao 266237, China; xf.chen@sdu.edu.cn or chen_smiling@163.com

Content

Figure S1 The total energy dependent on the lattice volume of the designed and dehydrated alkaline-earth metal cation-exchanged chabazite sieves (CHA-M). (a) CHA-Be, (b) CHA-Mg, (c) CHA-Ca, (d) CHA-Sr, and (e) CHA-Ba. 2

Table S1. The fractional coordinates of CHA-Be, CHA-Mg, CHA-Ca, CHA-Sr, and CHA-Ba... 3

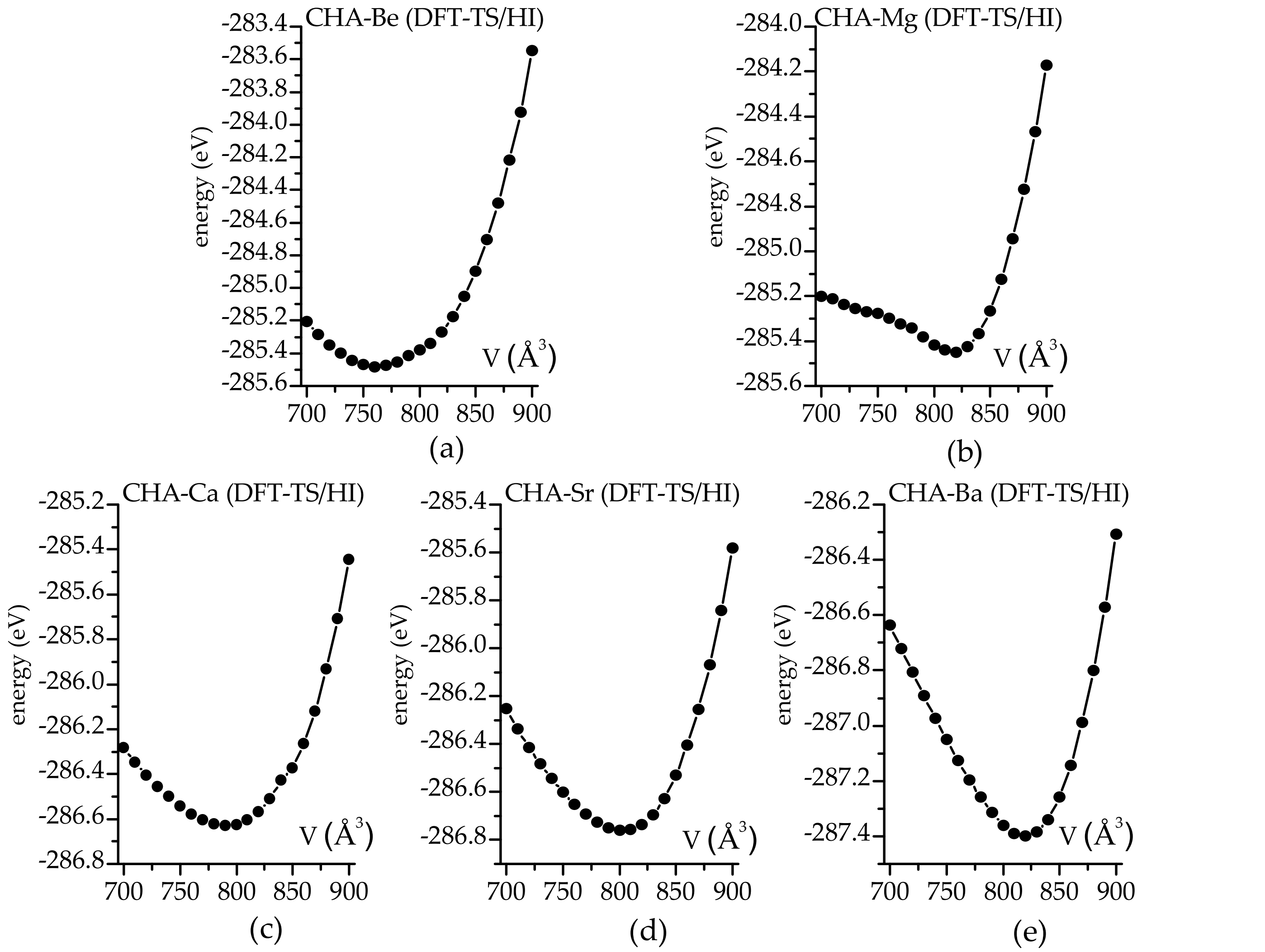

Figure S1 The total energy dependent on the lattice volume of the designed and dehydrated alkaline-earth metal cation-exchanged chabazite sieves (CHA-M). (a) CHA-Be, (b) CHA-Mg, (c) CHA-Ca, (d) CHA-Sr, and (e) CHA-Ba.

Table S1. The fractional coordinates of CHA-Be, CHA-Mg, CHA-Ca, CHA-Sr, and CHA-Ba.

#1. CHA-Be (Si10Al2O24Be1)

0.1332099817202064	0.3492665945821400	0.8970572909766830
0.9249035488536990	0.1599439163600849	0.3581944466522415
0.3847298224972349	0.9183861530586981	0.1568751699805517
0.7079572000145049	0.9289970414729254	0.1564152258318003
0.9482688020428114	0.7276018112375411	0.1469386091725298
0.1486891166475743	0.9083479207523126	0.6910649713581591
0.6957405897794757	0.1545345312986584	0.9189509924923129
0.3667234232595433	0.1138609281843159	0.9067918232317282
0.1479623589881101	0.9138607938952248	0.3716698559442975
0.9151164624874326	0.3579442934799815	0.1088766842475977
0.9224444021922906	0.1806303207557463	0.6787148378072629
0.1748185025648183	0.7416309238307477	0.9474971337194447
0.3328142187486023	0.7731746634998231	0.0490805668137426
0.0374365833395913	0.3646478794836909	0.7416111292016510
0.8016554870078920	0.0449863237541308	0.2710372605043929
0.7569857372306572	0.2962593323511484	0.0357554134586238
0.0351392980293852	0.7718609324223920	0.3010541273695821
0.2869264984882776	0.9634383527433954	0.8076373656447871
0.1933472397629075	0.8753921683116062	0.5338428836384352
0.5264682135460532	0.1671413341160957	0.8667965150830739
0.9411855611161215	0.5428886146750145	0.1091343422162367
0.8772790953480865	0.2111809989909901	0.5076327193639045
0.5500922835401525	0.9049515715370688	0.2122964052841070
0.1724313266955519	0.5371833576492548	0.9074069918063898
0.2720565085581228	0.2597079512335156	0.8788740574004734
0.0751146736605293	0.0767432726612967	0.3704671099337418
0.9488639409059871	0.3119496656561651	0.2671328907820296
0.3678246266081189	0.0763213012290080	0.0702094589446105
0.2937402661202242	0.9296402079943604	0.2929348050753404
0.0367425274710556	0.2848292873677423	0.0149134881248330
0.7825371996047821	0.7670799052498367	0.1488830844294071
0.0311548996827327	0.0284269903820089	0.7030655112316211
0.7858192573808296	0.1616437552723724	0.7861032088062672
0.0331877813759505	0.8110995211522152	0.0274292291765050
0.1064756269564313	0.7317206897213140	0.7527366389556960
0.7017902989530498	0.9939865662724898	0.0004247181724537
0.0872047529750262	0.5496702521663437	0.7572893884186556

#2. CHA-Mg (Si10Al2O24Mg1)

0.1583021416175896	0.3301671076007366	0.8843263151092771
0.9471011152279729	0.1269945159217372	0.3450191069806365
0.4043558894072419	0.8964221914610633	0.1306142409743245
0.7288318785832786	0.9062782565342715	0.1382071321037870
0.9597606635587184	0.6902789549618049	0.1356927760620934
0.1856631536729054	0.8918684211929531	0.6740525089327321
0.7221070485809093	0.1287309708555497	0.9100560891231382
0.3937291531493514	0.0992882324194824	0.8916186412801466
0.1698097456787977	0.8892494559882849	0.3513624145696923
0.9381841557238459	0.3352860598664265	0.1091843553191367
0.9469562824360338	0.1379281652301501	0.6684584185521842
0.1942283489791521	0.6970634479622646	0.9252926458802762
0.3528628963485261	0.7594649461618204	0.0172754236613812
0.0592644551967609	0.3088830692292746	0.7285228510095436
0.8239806279807169	0.0180725152700916	0.2544811275996679
0.7867997568539877	0.2632530602628691	0.0291608532575864
0.0539873622808358	0.7534797426436199	0.2844786825780758
0.3278257881753959	0.9674558692479849	0.7724324875042825
0.2219120215197279	0.8521394142141503	0.5120904763665948
0.5548493111785575	0.1554173373458170	0.8626575111740777
0.9376331105511682	0.5118428281938421	0.1341001055025757
0.8995810746161510	0.1746608685701290	0.4971164587864081
0.5697866231751263	0.8837371263054195	0.1908590493352023
0.2011127463997227	0.5061182503308501	0.8792943139946772
0.2978652088582621	0.2402023866797336	0.8783693183472607
0.0977668827784939	0.0461772690049358	0.3547654269422793
0.9743445531387991	0.2758382164559308	0.2625995804416590
0.3888947548456372	0.0491867173338889	0.0510866813453532
0.3101762667198571	0.9016873181819278	0.2654104932959385
0.0684318493375429	0.2903730694906059	0.0137021673910525
0.7998535645546809	0.7486868830073661	0.1297059133849316
0.0530833625364906	0.9902864523619144	0.6851449206385354
0.8101808929378436	0.1267217323082832	0.7755165051627628
0.0418909901459656	0.7373096133109485	0.0032345753087171
0.1541252998835816	0.7260319871778265	0.7365008072771175
0.7234896762401988	0.9723486278235569	0.9844359469325781
0.1046162455592565	0.5151096849018515	0.6854828257471368

#3. CHA-Ca (Si10Al2O24Ca1)

0.1534657632481142	0.3762866755861509	0.8818289859834962
0.9402192938597125	0.1577473777212361	0.3460308393431291
0.3921965758491197	0.9231764325004050	0.1305324062842033
0.7215044655987697	0.9431593167759260	0.1418296040515585
0.9468683993255951	0.7101344205544748	0.1484576645516000
0.1751307833744420	0.9303444631574465	0.6815561951751405
0.7134840395226476	0.1640551414722538	0.9141536289242254
0.3808072598224754	0.1348214712316107	0.9001875829407382
0.1597025982580433	0.9139284224820230	0.3567951170956434
0.9292640175919971	0.3764972910337931	0.1174097918264252
0.9372238727427273	0.1740427629811450	0.6716628266753162

0.1860832049256800	0.7166140551633049	0.9205814772912788
0.3405088337771787	0.7898839366909129	0.0159607682003298
0.0475689923541580	0.3372261020384002	0.7347420155792861
0.8066048005569328	0.0565254674258711	0.2636736208970021
0.7852049251427857	0.2961943081379843	0.0303182119646621
0.0459615105201792	0.7760456416492616	0.2950485510891809
0.3190586240450841	0.0112948063779328	0.7731130632903742
0.2127461664572436	0.8812673489083878	0.5190223552043776
0.5440709185891635	0.1927352605349668	0.8770729470908236
0.9004338587796994	0.5413069528565728	0.1721104765898360
0.9004414540245733	0.2195249918688305	0.4989328167566143
0.5597352966822911	0.9127274844998183	0.1885051410962575
0.2134593774128177	0.5388541789001309	0.8500350237946037
0.2833372662337155	0.2725415929567347	0.8945777073796179
0.0841005510962276	0.0660567501548996	0.3553417921059392
0.9792999663784130	0.2973066878430757	0.2578252213200827
0.3729209812227765	0.0754369270983020	0.0563269451166732
0.2999792444818681	0.9243535919470105	0.2687243078317181
0.0660140255653374	0.3765765984845046	0.0211017863117391
0.7985952186887957	0.7918304426770462	0.1339168258433006
0.0404147986897954	0.0265494227132876	0.6840024052601805
0.7932832309253826	0.1595247661941741	0.7724877105438068
0.0335318323629750	0.7227418525943108	0.0109191934903805
0.1403066037986562	0.7721641243701498	0.7457915652705793
0.7204106262632095	0.0108418987750696	0.9887892330228141
0.0938996275605604	0.5464069400774036	0.6309016988454275

#4. CHA-Sr (Si10Al2O24Sr1)

0.1531670449023323	0.3547522990936258	0.8816715204114658
0.9365226462517882	0.1329151113742313	0.3463307543918006
0.3894870710435043	0.8961970438126770	0.1283281498819306
0.7212974401038963	0.9215124742357546	0.1468303232764399
0.9491841317951000	0.6827658239942380	0.1531713898199030
0.1770159147435493	0.9076558959521819	0.6828408895137486
0.7148392045732095	0.1376219831177679	0.9136793312339861
0.3801586254982112	0.1104012333790720	0.9004454756069720
0.1592097547463709	0.8836372223069020	0.3574528550686793
0.9314178142592482	0.3520106121560076	0.1162312360254560
0.9360327818203658	0.1510267998685890	0.6732122066426882
0.1840371473413143	0.6897443706399926	0.9184238742846347
0.3382827781896864	0.7634326326167624	0.0142373953378012
0.0395632798893857	0.3127262810991738	0.7416453800757381
0.7943106744221069	0.0402048687581456	0.2709028374515583
0.7910164381414830	0.2719944490372868	0.0239429338289483
0.0527515476447604	0.7385202153755088	0.3013307395077689
0.3220850046851638	0.9878344938951003	0.7718248412156896
0.2155475695321414	0.8561773855189614	0.5203195805117460
0.5446197783223781	0.1674829115754619	0.8824270175824864
0.8966946661780639	0.5149484952632690	0.1741692538842230
0.9066622571521350	0.2031628199065452	0.5003141986984829
0.5577435504288957	0.8851032124995584	0.1853703938792108
0.2131828029236473	0.5142063841473998	0.8452714720305678
0.2825940499729072	0.2482879488800691	0.8918955916590576
0.0732247945954612	0.0300048720092718	0.3507035109939594
0.9798266103514806	0.2693096803527411	0.2558114455262697
0.3684967030410675	0.0492877667576224	0.0558512643676181
0.2979608077393436	0.8976339518813248	0.2676391153678779
0.0729784551721764	0.3587443111945703	0.0267644231953739
0.8059018250917376	0.7740558135475553	0.1473797698843100
0.0426961641840364	0.0056869340176178	0.6812115701036134
0.7876558158286429	0.1276186741731777	0.7674424572166458
0.0336665249801342	0.6956410567643587	0.0143007843322778
0.1379415233092658	0.7524845632817119	0.7477031923754609
0.7265191900501762	0.9871321478188477	0.9928378325630618
0.0821975829279964	0.5202104782669679	0.6029599609972465

#5. CHA-Ba (Si10Al2O24Ba1)

0.1190176003537999	0.3521597661337310	0.9057330285399061
0.9069901625809678	0.1382412336154815	0.3540175976475979
0.3521669391924007	0.9101710418131503	0.1443111746652193
0.6754721395531931	0.9099455731642081	0.1362450393857841
0.8975802286164907	0.6875945082999593	0.1398580504815357
0.1137565033863623	0.9001436847074231	0.6841586748076196
0.6642199204119024	0.1284844363519539	0.9127260680462399
0.3367722924865930	0.1249863127918118	0.9219550999534007
0.1266516896041949	0.9098498464097631	0.3639334897364037
0.8948587904630827	0.3572537826191962	0.1313063567948376
0.8840011333817301	0.1237759173082438	0.6787557961067066
0.1275236557737500	0.6880188636394209	0.9148893229405246
0.2757937101454715	0.7788573463088468	0.0265076260390700
0.0124096251975345	0.2705114386519938	0.7635235006468548
0.7810040448758429	0.0286696785438565	0.2540940648812438
0.7552320132327921	0.2589815477604418	0.0353670595569895
0.0061671979601599	0.7767075172402969	0.2812482810436521
0.2383997238582154	0.0081021379945270	0.7961713295010924
0.1733031234574653	0.8719409218191032	0.5268477373904403
0.4976928647502561	0.1656893152170511	0.8792809198663392
0.8613363350461043	0.5219498908430111	0.1826027384929603
0.8621272323798834	0.1936873174963551	0.5097817183194167
0.5204376110792879	0.8855065845002983	0.1953626648125208
0.1602718018718647	0.5102499979747961	0.8561547673738374
0.2640165439162203	0.2750665630775444	0.9400734928474890
0.0586373234332953	0.0651096640943862	0.3679293083961284
0.9391034086377346	0.2924381245590197	0.2853844849815346

0.3490372938610591	0.0649226471680322	0.0783015963868507
0.2728032634799931	0.9240474523744666	0.2892785800115334
0.0378562137641012	0.3611150628758324	0.0498913519576405
0.7444549898202055	0.7572580023313478	0.1240599923568197
0.9606888879761115	0.9635513122296970	0.6639033719300684
0.7328992216612207	0.1178216779051411	0.7656738752850103
0.9687038453047236	0.6851046280564077	0.9938031885866891
0.0927987295634694	0.7401183002647116	0.7388060136375714
0.6620822191915821	0.9744111787664096	0.9819433305848762
0.0179347805012497	0.4738727095051587	0.5812032218590062
